# Insights into the Prognostic Value of Telomere Length in Childhood Acute Lymphoblastic Leukemia

**DOI:** 10.3390/life15101537

**Published:** 2025-10-01

**Authors:** Elena Vakonaki, Iordanis Pelagiadis, Stella Baliou, Manolis N. Tzatzarakis, Athanasios Alegakis, Ioanna Lygerou, Persefoni Fragkiadaki, Maria Stratigaki, Nikolaos Katzilakis, Aristidis Tsatsakis, Eftichia Stiakaki

**Affiliations:** 1 Laboratory of Toxicology, Medical School, University of Crete, 71003 Heraklion, Greece; 2 MSc Program “Hematology-Oncology of Childhood and Adolescence”, School of Medicine, University of Crete, 71003 Heraklion, Greeceefstel@uoc.gr (E.S.); 3 Department of Pediatric Hematology-Oncology and Autologous Hematopoietic Stem Cell Transplanta-tion Unit, University Hospital of Heraklion and Laboratory of Blood Diseases and Childhood Cancer Bi-ology, School of Medicine, University of Crete, 71003 Heraklion, Greece; 4 Universidad Ecotec, Km. 13.5 Samborondón, Samborondón EC092302, Ecuador; 5 Sechenov IM First State Medical University, Moscow 119991, Russia

**Keywords:** acute lymphoblastic leukemia (ALL), childhood, telomere length

## Abstract

Background: Although telomere length maintenance is a common characteristic of hematological malignancies, the role of telomere length as a prognostic factor to stratify acute lymphoblastic leukemia (ALL) patients depending on their risk of relapse remains elusive. Methods: This knowledge gap motivated us to examine telomere length values in children with ALL at the time of diagnosis and after treatment using quantitative polymerase chain reaction (qPCR) (*n* = 35). To achieve high-resolution precision and cell specificity, a quantitative fluorescence in situ hybridization (qFISH) technique was developed (*n* = 5). Results: The results demonstrated statistically significant evidence of telomere shortening in the lymphoblasts of children with ALL but not in the lymphocytes of children after remission following treatment. Our findings also suggested a significant association between telomere shortening and a high risk of relapse disease. Last but not least, our preliminary results showed a trend that telomere shortening was more pronounced in children with B-ALL compared to those with T-ALL in a non-significant manner. Conclusions: Consequently, the current study provides preliminary insights into the potentially substantial prognostic value of telomere length in the progression of pediatric ALL, with the possibility of predicting treatment response. To clarify the application of telomere length as a possible biomarker for disease progression and treatment response in children with ALL, the telomere length values of additional participants need to be examined in further studies.

## 1. Introduction

At the ends of chromosomes, telomeres consist of repeated DNA sequences of 5′-TTAGGG-3′ that serve as protective caps associated with the shelterin protein complex [1]. Each cycle of cellular division can result in the shortening of telomere length due to end-replication problem [2]. Progressive telomere shortening results from a non-replicable single-stranded overhang at the 3′ end provided by each cell division [2]. Accelerated aging is characterized by telomere shortening. Indeed, telomere shortening is connected with many aging hallmarks including genomic instability, inflammation, oxidative stress, epigenetic reprogramming, stem cell depletion, and mitochondrial dysregulation [2]. On the molecular setting, the critical short length of telomeres prevents their protection by the shelterin protein complex, and for this reason, telomeres can be recognized as double-strand breaks (DSBs), thus activating the DNA damage response (DDR), resulting in cellular senescence [3]. Additionally, in cancer, unexposed telomeres are common, leading to end-to-end chromosomal fusions and other chromosome abnormalities, thereby contributing to the increased genome complexity observed [3].

Telomeres can serve as crucial indicators of cellular health, offering insights into a wide range of physiological and pathological conditions [4]. Since telomeres are crucial for assessing an individual’s cellular health, short telomere length values are frequently associated with age-related conditions including diabetes, osteoporosis, autoimmune diseases, neuro-degenerative diseases, cardiovascular diseases, and certain types of cancer [5,6,7,8,9,10,11]. A ribonucleoprotein enzyme known as telomerase creates telomeric repeats at the ends of chromosomes using a small portion of RNA as a template to halt chromosomal shortening [12].

According to research, shorter telomeres may increase the risk of leukemia progression [13] and telomere length is often considered a prognostic indicator in acute lymphoblastic leukemia (ALL) [14]. ALL cases with cytogenetic defects typically exhibit an increased telomere shortening rate, suggesting that telomere length can predict the aggressiveness of ALL [15].

Acute lymphoblastic leukemia (ALL) is the most common neoplasia in children, covering approximately 25–30% of all childhood cancer cases. Clonal evolution of precursor lymphoid cells plays an essential role in the development of ALL, which can be classified into two main subtypes, B-lymphoblastic (B-ALL) and T-lymphoblastic leukemia (T-ALL), based on the lineage of the precursor cells, even though the WHO5-ICC hematopoietic classification now mandates a further, genetic emphasis in the nomenclature. Notably, T-ALL is less commonly observed in children, accounting for approximately 15% of cases. The application of structured multidrug treatment protocols, patient risk stratification, and the introduction of further knowledge about the genetics of the disease have enabled an overall survival rate of over 90% [16]; nevertheless, 20% of treated children may still experience relapse, and 10% of these remain untreated [17,18]. Both B- and T-ALL exhibit distinct characteristic chromosomal alterations, including aneuploidies, chromosomal rearrangements, and mutations [19].

In current treatment protocols, patients are stratified into different treatment intensity groups based on various predictive factors of relapse. Morphology, immunophenotype, molecular genetics, and mainly the response to initial treatment assessed with minimal residual disease (MRD) contribute to the prognostic armamentarium, thereby providing a precise diagnosis and effective stratification of ALL, both in adults and in children [19,20]. Regarding the prognostic parameters of ALL, historically, the age and the white blood cell count at diagnosis were recognized as two significant prognostic factors in both T- and B-ALL. In this context, children between 1 and 10 years of age with a white cell count below 50,000/mm were considered to have a more favorable prognosis [19,20]. Overall, two-thirds of B-ALL patients are between the ages of 1 and 10, and they typically present with an initial white blood cell count (WBC) of less than 50,000/mm^3^. B-ALL is generally considered more favorable compared to the T counterpart [19,20]. Children who exhibit an adequate response to treatment, as assessed by the measurement of MRD at specific time points, have a favorable prognosis [19,21].

More recently, the field of cytogenetics has gained greater importance in determining disease outcome and risk stratification. For instance, hyperdiploidy, characterized by the presence of more than 50 chromosomes or a DNA index exceeding 1.16, is recognized as a favorable genetic factor and is the most common chromosomal abnormality in pediatric ALL, accounting for about 20% of cases [20]. Another frequent subtype, found in 20–25% of childhood ALL, involves the *ETV6::RUNX1* fusion gene, which arises from the cryptic translocation of t (12;21) (p13;q22). Moreover, recent evidence indicates that intragenic deletions of the *ERG* gene are linked to better prognoses in pediatric B-cell ALL [22]. Interestingly, the presence of *ERG* deletions may influence the prognostic significance of *IKZF1* gene abnormalities.

Hypodiploidy has been linked to poorer prognoses in pediatric ALL, with this feature present in about 1% of cases. Low hypodiploidy frequently coincides with *TP53* gene changes, and approximately half of these are inherited germline mutations. Alterations involving the *KMT2A* gene, formerly known as *MLL*, are detected in around 5% of childhood ALL, with a higher prevalence among infants; these genetic rearrangements are strongly associated with more aggressive disease and adverse clinical outcomes [20]. Pediatric ALL cases with the rare *TCF3::HLF* fusion, caused by a t (17;19) (q22;p13) chromosomal change, make up about 1% of diagnoses and have recently been recognized for their particularly unfavorable outcomes [23]. Conversely, the *TCF3::PBX1* gene fusion, resulting from the recurrent t (1;19) (q23;p13) translocation, also occurs in nearly 5% of young patients but is linked to intermediate treatment results [24].

A specific chromosomal abnormality known as intrachromosomal amplification of chromosome 21 (iAMP21) is present in approximately 2% of children with ALL. iAMP21 is defined by either three or more extra *RUNX1* copies on a single chromosome, as seen in metaphase FISH, or at least five *RUNX1* FISH signals per interphase nucleus. This particular genetic anomaly is more common among adolescents and young adults, typically between ages 9 and 11, and is associated with a greater risk of a poor outcome if treated with less intensive protocols [20]; however, intensifying therapy can yield better prognoses. Moreover, the *BCR::ABL1* fusion gene, created by the t (9;22) (q34;q11) translocation, is detected in about 2% of pediatric ALL cases—less often than in adolescents and young adults—and has long been recognized as a marker of higher-risk disease under conventional therapies [25].

Several other rare but clinically significant genetic alterations are recognized in pediatric acute lymphoblastic leukemia (ALL), each associated with distinct prognostic implications. Among these, gene fusions arising from chromosomal translocations play a critical role in risk stratification. For instance, the *TCF3::HLF* fusion, which results from a t (17;19) (q22;p13) rearrangement, occurs in a very small proportion—close to 1%—of childhood ALL cases, yet it is now well established that this alteration confers a particularly aggressive disease course and poor survival outcome [23]. In contrast, *TCF3::PBX1* is generated by the more common t (1;19) (q23;p13) translocation, observed in about 5% of children diagnosed with ALL. Cases harboring *TCF3::PBX1* are generally considered to carry an intermediate risk and respond more favorably to therapy compared to *TCF3::HLF* rearrangements [24]. Another molecular event with significant impact on patient management is the generation of the *BCR::ABL1* fusion gene, caused by the t (9;22) (q34;q11) translocation. In the pediatric setting, this fusion is relatively infrequent, occurring in close to 2% of cases—a rate that increases with age. The presence of *BCR::ABL1* has been associated with resistance to standard therapies and has historically signified a group with poorer prognoses, though outcomes have improved with targeted tyrosine kinase inhibitor therapies [25].

In pediatric B-ALL, mutations affecting the Ikaros gene (*IKZF1*) occur in roughly 15–20% of cases and are notably more frequent among patients who also harbor the *BCR::ABL1* fusion. Numerous studies have linked *IKZF1* alterations to poorer clinical outcomes and an increased risk of disease relapse [26]. Recent research has identified a subgroup termed *IKZF1plus*, characterized by *IKZF1* deletions coexisting with deletions in *CDKN2A*, *CDKN2B* (specifically homozygous deletions), and *PAX5* or deletions within the *PAR1* region, resulting in the *P2RY8::CRLF2* fusion, while lacking *ERG* deletions. This subset is associated with slower clearance of minimal residual disease (MRD) and a worse prognosis and has been integrated into contemporary risk classification systems [27].

Overexpression of *CRLF2* on leukemic lymphoblasts can result from multiple genetic mechanisms, including translocation involving the immunoglobulin heavy chain locus (*IGH::CRLF2*), an interstitial deletion in the *PAR1* region that produces the *P2RY8::CRLF2* fusion, or less commonly, point mutations such as *F232C* within *CRLF2*. These *CRLF2* rearrangements predominantly appear in *BCR::ABL1-like* and Down syndrome-associated ALL and are generally linked with unfavorable outcomes, primarily when accompanied by *IKZF1* mutations [20].

The *BCR::ABL1-like* (Ph-like) subgroup accounts for approximately 15% of childhood ALL cases and features a gene expression profile closely resembling that of Ph-positive ALL despite lacking the Philadelphia chromosome. Over half of Ph-like ALL cases harbor *IKZF1* alterations. This subgroup is frequently characterized by gene fusions and mutations that activate tyrosine kinase signaling pathways, such as rearrangements in *ABL* class genes (*ABL1*, *ABL2*, *PDGFRA*, *PDGFRB*, *FGFR*), *JAK-STAT* pathway components (*CRLF2*, *EPOR*, *JAK1-3*, *TYK2*, *SH2B3*, *IL7R*), and others including *FLT3*, *NTRK3*, *LYN*, and *PTK2B*. These molecular alterations lead to challenging clinical scenarios, raising the potential for targeted therapies including ABL class tyrosine kinase inhibitors and JAK inhibitors to improve outcomes [28].

In the context of T-ALL, activating mutations of *NOTCH1* are seen in over half of the cases, whereas mutations in *FBXW7*—a ubiquitin ligase regulating *NOTCH1* degradation—occur in roughly 10–15%, often resulting in sustained *NOTCH1* signaling. While these mutations generally correlate with favorable prognosis and lower MRD levels, their independent predictive value remains uncertain and may depend on the absence of other mutations such as *RAS* or *PTEN*. Approximately half of T-ALL cases involve chromosomal translocations affecting T-cell receptor genes, frequently involving transcription factors like *TAL1*, *TAL2*, *LYL1*, *OLIG2*, *LMO1/2*, *TLX1/3*, *NKX2* family members, *HOXA* genes, *MYC*, and *Myb*. Additionally, cryptic rearrangements involving ABL1—particularly *ABL1/NUP214* fusions with episomal amplification—are observed. Alterations impacting cell cycle regulation are common as well, with *CDKN2A* deletions identified in about 70% of patients [29]. Despite these diverse genetic features, T-ALL-associated abnormalities have yet to be fully incorporated into current risk stratification protocols. These prognostic factors in ALL, including Ph-like ALL and IKZF1 alterations, have been thoroughly studied and characterized by groups like COG and DCOG [30,31,32,33,34,35,36]. The comprehensive molecular characterization highlights the complexity and heterogeneity of pediatric ALL, underscoring the importance of integrating genomic data into risk assessment and therapeutic decision-making.

From a therapeutic perspective, treatment protocols such as the BFM (Berlin–Frankfurt–Münster)-based protocols have been proven to be effective in setting the disease in remission, minimizing the chances of relapse. Typically, such treatment protocols consist of specific phases—remission induction, consolidation, re-induction (delayed intensification), and maintenance treatment—while a special central nervous system (CNS) prophylactic or therapeutic treatment is employed. The ALLIC-BFM 2009 stratifies patients into the following risk group of relapse: (a) standard risk (SR): initial WBC < 20,000/µL, age ≥ 1 yr–≤6 yr, peripheral blood day 8 < 1000 blasts/µL, and flowcytometry MRD on day 15 < 0.1%—all criteria must be fulfilled; (b) high risk (HR): (i) peripheral blood on day 8: ≥1000 blasts/µL, (ii) flowcytometry MRD on day 15 >15% or MRD on day 33 >10%, (iii) translocation t (9;22) [BCR/ABL] or t (4;11) [MLL/AF4] and hypodiploidy ≤ 44—at least one criterion must be fulfilled; (c) intermediate risk (IR): all patients who are not stratified into SR or HR.

A variety of anti-cancer medications, such as alkylating agents, topoisomerase inhibitors, and platinum drugs, as well as radiation, cause oxidative stress, resulting in the increased generation of DNA double-strand breaks. During oxidative stress conditions caused by therapy, telomeric DNA is more susceptible than other genomic areas due to its G-rich nature. As a result, leukemic cells present rapid telomere erosion, which causes them to enter senescence or apoptosis [37,38].

Considering the limited data in the field, our aim was to investigate the prognostic role of telomere length as a biomarker for predicting the response of children with ALL to treatment.

## 2. Materials and Methods

This study was conducted in accordance with the Declaration of Helsinki, and the protocol was approved by the University Hospital of Heraklion’s Research Ethics Committee (112/08.09.2021). All of the study’s samples were anonymized, and the General Data Protection Regulation (GDPR; URL (assessed on 4 September 2025) https://gdpr-info.eu/) of the European Union was followed when handling personal data.

Bone marrow (BM) samples were collected from children with a diagnosis of acute lymphoblastic leukemia (ALL). All patients received treatment according to the International Berlin–Frankfurt–Münster Study Group 2009 protocol (ALLIC-BFM 2009) (URL (assessed on 4 September 2025) https://www.bialaczka.org/wp-content/uploads/2016/10/ALLIC_BFM_2009.pdf). The samples from each patient were collected at two different time points: one at the diagnosis of the disease and the other during complete remission, as assessed by the MRD measurement. Following the manufacturer’s recommendations, genomic DNA was extracted using the DNeasy Blood & Tissue Kit (Qiagen, Hilden, Germany). DNA was extracted using the QIAamp DNA Mini kit (Cat. No. 51306, QIAGEN, Hilden, Germany). According to the instructions, the Relative Human Telomere Length Quantification qPCR Assay Kit (ScienCell, Cat. No. 8918, Carlsbad, CA, USA) was used to examine telomere length values. The average telomere length at each chromosome end was calculated after the qPCR reaction using an Agilent Technologies Stratagene Mx3005P machine (Agilent Technologies, Santa Clara, CA, USA), following the kit’s instructions.

In addition, the metaphase quantitative fluorescence in situ hybridization (qFISH) technique was developed in our lab [28]. Lithium heparin tubes (2 mL) were used to collect bone marrow samples. Five milliliters of RPMI-1640 lymphocyte culture medium supplemented with 10% fetal bovine serum, 1% L-glutamine, 1% penicillin, and 1% streptomycin (Sigma-Aldrich, St. Louis, MO, USA) was used to culture bone marrow cells for seventy-two hours at 37 °C and 5% CO_2_ to achieve confluency levels. To arrest chromosomes at the metaphase stage, 10 µg/mL colcemid was applied to each cell for the production of metaphase chromosomes. The fixation protocol followed this step. The Q-FISH protocol was carried out on cells with chromosomes at the metaphase stage utilizing a Peptide Nucleic Acid (PNA) probe (Eurogentec). Then, images were captured using a fluorescence microscope, and telomere lengths were measured with specialized software [26]. An Axio Imager M1 microscope (Carl Zeiss, Oberkochen, Germany) equipped with a CoolCube 1 CCD camera (MetaSystems, Altlussheim, Germany) and a 63× objective magnification was used to capture images of cells with chromosomes at the metaphase stage. ISIS software, version V 5.9.1 CM (MetaSystems, Altlussheim, Germany) was used to accurately measure telomere length. In particular, 10 metaphases via the qFISH method per patient were used for determining median telomere length and the percentage of short and critical short telomeres in every patient. High-quality images were collected and studied under a microscope, excluding cells with close metaphases in which overlapping chromosomes appeared. To ensure a reliable quantitative assessment of telomere length across the different samples, two calibration levels were employed. First, photos of fluorescent beads (orange beads, 0.2 µm in size, Thermo Fisher Scientific (Waltham, MA, USA)) were taken before capturing the images of the samples to account for daily changes in lamp alignment and intensity. The ISIS program (MetaSystems, Altlussheim, Germany) was used to evaluate the fluorescence intensities of the telomeres and beads. Second, the mean of the L5178Y-S intensity values, which served as a reference value for every experiment, was used to normalize the telomere fluorescence intensity values across slides. L5178Y S cells (cat. no. 93050408; Culture Collections, Public Health England, Salisbury, UK) with an established and reported telomere length were used to transform telomere fluorescence values into kilobases.

Telomere length, age of onset, and current age were used as the continuous variables expressed in the form of mean and standard deviation, while all other study variables were qualitative or quantitative discrete variables and they were expressed in the form of counts and % frequencies. Differences in means were examined using independent samples t-test for two-group comparisons or paired samples *t*-test when comparison was performed during the diagnosis and remission period. IBM SPSS Statistics 24.0 was used for statistical analysis and a = 0.05 was set as the level of significance.

## 3. Results

In this study, a total of 35 children were included, with a mean age of 6.4 ± 4.1 years. The current age of the children at the time of statistical analysis (based on December 2021) was 11.8 ± 3.5 years (Table 1). Of the total samples involved in this study, 62.9% (*n* = 22) were boys, while the remaining 37.1% (*n* = 13) were girls (Table 1).

Children were categorized according to ALL subtype. Based on the children’s immunophenotype, 88.6% of children (*n* = 31) had B-ALL, which is the most common type in childhood, while 11.4% of children (*n* = 4) had T-lineage.

All patients underwent treatment according to the ALLIC-BFM 2009 protocol. As specified by the treatment protocol guidelines, 34.3% of children were assigned to the high-risk (HR) group, while the remaining 65.7% were categorized as intermediate risk (IR). No patient fulfilled the prerequisites to be allocated to the standard risk (SR) group. Following treatment, a total of three children with ALL relapsed (*n* = 3/35), while the rest of the group remained alive without recurrence of disease. Concerning the patients’ karyotypes, data were available for only 33 of 35 patients. Euploidy was observed in 63.6% (*n* = 21/35), while the rest presented hyperdiploidy (Table 2).

A total of 20% of children had either TEL-AML1 translocation or cyclin-dependent kinase inhibitor 2A-2B (CDKN2A-CDKN2B) translocation. A small percentage (2.85%) had a deficiency in the cyclin-dependent kinase inhibitor 2A (CDKN2A) tumor suppressor, and 14.28% had a loss of the paired box protein 5 (PAX5) transcription factor.

**Figure 1:**
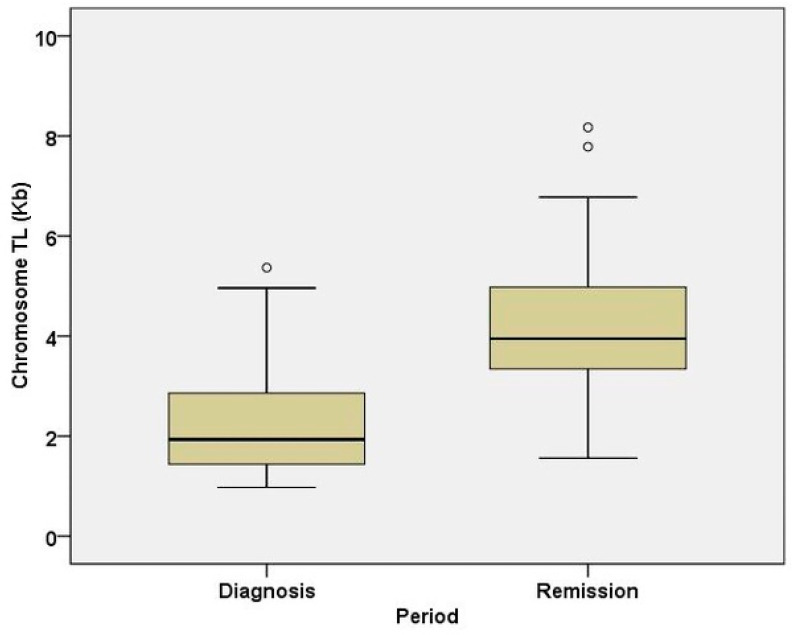
The distribution of telomere length values in children with acute lymphoblastic leukemia (ALL) at diagnosis and at the time of remission is shown in the graph. A statistically significant difference between the values is observed based on the paired *t*-test (*p* < 0.001). Abbreviations: telomere length (TL).

Initially, the qPCR results showed that the average telomere length of lymphocytes during remission is increased compared to that of lymphoblasts at diagnosis, highlighting a statistically significant positive relationship between average telomere length and responsiveness to treatment in children with ALL (Figure 1). Moreover, it is important to emphasize that a shorter telomere length was measured in leukemic lymphoblasts of children with ALL compared to the telomere length measured in lymphocytes during remission in these children. Therefore, the observed difference reflects a comparison between malignant and non-malignant cell populations, rather than telomere elongation within the same cells. In addition to this, ALL is a cancer that affects the bone marrow’s immature lymphoid precursors, or lymphoblasts. These blasts inhibit the formation of healthy blood cells, overpopulate the marrow, and multiply abnormally. These leukemic lymphoblasts constitute the main population of hematopoietic cells in the bone marrow (and frequently in peripheral blood) upon diagnosis because of the clonal growth [14]. On the contrary, the morphological definition of remission in ALL, particularly following induction chemotherapy, is the recovery of normal blood counts and the reduction in leukemic blasts in the bone marrow to below a certain threshold (often less than 5% blasts). [14].

To sum up, by definition, ALL clone consists of lymphoblasts as opposed to normal lymphocytes, which are seen in cases of remission.

In particular, the majority of ALL children receiving therapy tend to have a higher average telomere length (4.18 ± 1.54 kilobases) than that seen in ALL children at diagnosis (2.18 ± 1.04 kilobases) (Figure 1). As a result, the treated ALL group showed a statistically significant increase in average telomere length compared to the untreated ALL group (Figure 1).

**Figure 2:**
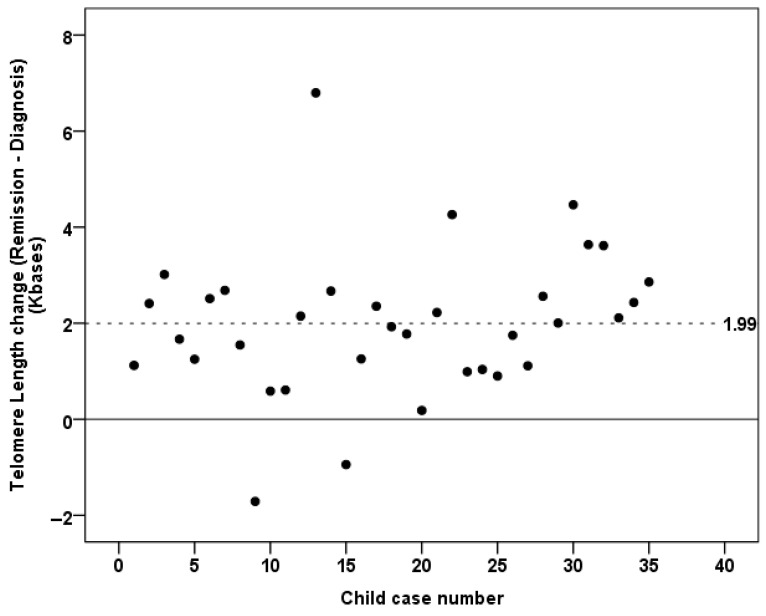
A clustered bar graph of the changes in average telomere length between remission and diagnosis of acute lymphoblastic leukemia (ALL) in children. The solid horizontal black line refers to zero difference; the dashed black line shows the mean TL of all measurements. An increase in TL was found among the lymphocytes in ALL children at remission.

**Figure 3:**
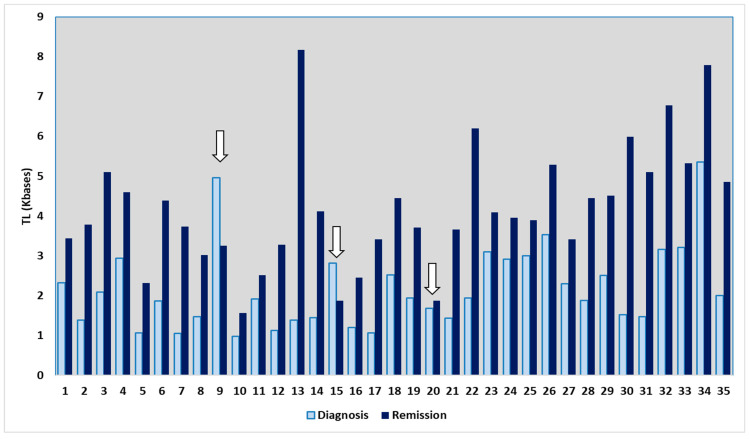
A clustered bar graph showing the changes in average telomere length between remission and diagnosis of acute lymphoblastic leukemia (ALL) in each child separately.

Then, the frequency of the average telomere length values was defined as the change in telomere length between children with ALL at remission and their initial telomere length at diagnosis (Figure 2). The mean frequency of the average telomere length was 1.99 ± 1.5 (Figure 2). In addition, it is essential to note that the average telomere length remained unchanged or reduced between the time of diagnosis and relapse in three children. In particular, no substantial variation in the average telomere length values was observed in one child with ALL who experienced relapse. The other two children exhibited a decrease in their average telomere length of 34.5% and 33.6%, respectively (Figure 2 and Figure 3).

In particular, the individual measurements for each patient are shown in Figure 3. The blue bars represent measurements taken at diagnosis, while the light blue bars show measurements during remission. In 91.4% (*n* = 32) of patients, the measurements indicate an increase in telomere length during remission. The exceptions are three cases marked with white arrows (cases 9, 15, and 20), the last of which involves a child who relapsed and showed no apparent change in length.

In addition to the above, the average telomere length was lower in the group with B-ALL (1.90 ± 1.67 kilobases) compared to in children with T-cell ALL (2.40 ± 1.08 kilobases) (Table 3). However, this difference was not statistically significant. According to their karyotypes, the average telomere length of untreated ALL children with 46 chromosomes was 1.61 ± 1.4 kilobases, which is statistically significantly shorter than that of children with more than 46 chromosomes (3.02 ± 1.78 kilobases) (*p* = 0.030).

**Figure 4:**
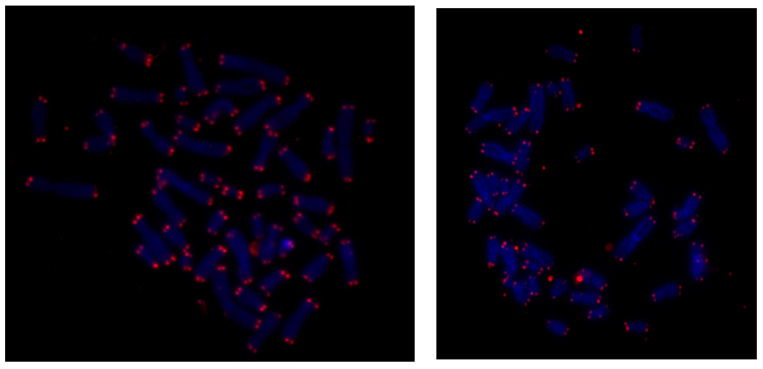
Quantitative fluorescence in situ hybridization (qFISH) images from two children with acute lymphoblastic leukemia (ALL). Fluorescence intensities (red marks) are roughly proportional to telomeric length (TL) and nuclei are presented by blue marks.

To strengthen our results regarding mean telomere length, in five patients, qFISH experiments were performed to evaluate the median telomere length in a cell in a chromosome-specific manner. In particular, the qFISH methodology provided preliminary data regarding the pronounced telomere shortening present in children with ALL (Figure 4).

**Figure 5:**
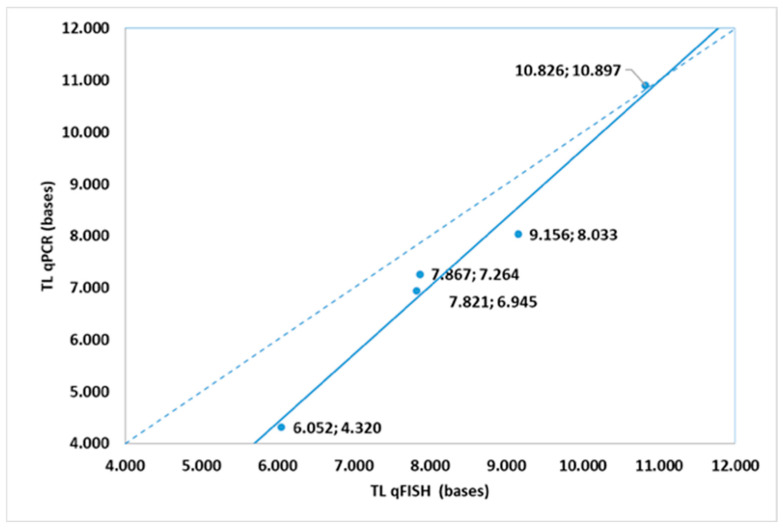
Measurements of telomere length in children with acute lymphoblastic leukemia (ALL) using two methods [quantitative fluorescence in situ hybridization (qFISH) and quantitative polymerase chain reaction (qPCR)]. The solid line represents linear correlation, while the dotted line indicates absolute consistency between the two methods. An underestimation of qPCR telomeric length method vs. the qFISH method was observed.

Last but not least, a novel comparison of the two methodologies used to evaluate telomere length values during ALL progression was performed. The statistical analysis showed that our qFISH experiments provided preliminary insights into the occurrence of telomere shortening in the lymphoblasts of ALL children at the time of diagnosis, demonstrating consistency between qPCR and qFISH techniques (Figure 5). There was a statistically significant correlation (*r* = 0.989, *p* < 0.001) in telomere length measurements in the indicative subsample of five children (*n* = 5) measured using qPCR and qFISH molecular biology methods (Figure 4).

## 4. Discussion

This study provides preliminary evidence regarding the translational implications of telomere length in children with acute lymphoblastic leukemia (ALL), highlighting the significance of telomere length as a potential prognostic marker for monitoring disease progression and therapy efficacy.

In particular, our qPCR results have shown that the average telomere length was greater in the lymphocytes derived from children with ALL who have achieved remission compared to that of lymphoblasts derived from ALL children at diagnosis, reinforcing the concept that telomere length can serve as a potential indicator of the effectiveness of therapeutic options in ALL children. The preliminary qPCR results have demonstrated that the telomere length of the lymphocytes of children with B-ALL at the time of remission was approximately twice that of the lymphoblasts at diagnosis, highlighting the predominance of normal lymphocytes with longer telomeres after treatment in ALL children who entered remission. To strengthen our results, we developed a quantitative fluorescence in situ hybridization (q-FISH) technique in our laboratory, providing preliminary insights suggesting that telomere shortening occurred in all children with ALL at diagnosis. The metaphase qFISH method identified short telomere length values in children with ALL in each cell and on each chromosome with high molecular precision, thus preventing inaccuracies in recognizing interstitial telomere areas across chromosomes [39]. Additionally, our qPCR results have provided preliminary evidence that children in the high-risk ALL group showed a greater telomere shortening rate than those with intermediate-risk leukemias, supporting the notion that the rate of telomere erosion can precede the aggressiveness of the disease. Moreover, the preliminary results derived from the qFISH technique confirmed the qPCR results.

Regarding the main preliminary finding of this study, the average telomere length of the lymphocytes of ALL children who experienced remission following treatment was greater than that derived from lymphoblasts of ALL children at diagnosis. Consistent with the preliminary findings of our study, numerous studies have supported the idea that telomere shortening is more pronounced in lymphoblasts than in normal lymphocytes in children with ALL. For instance, Borssén and colleagues have employed the qPCR technique, demonstrating that the average telomere length of blasts was significantly shorter in ALL children than in normal hematopoietic cells during remission [40]. Accordingly, the qPCR results support that extremely short length values may serve as a potential prognostic indicator for the progression of ALL [41]. In addition, short telomere length values can be primarily characteristic of extensive proliferation of leukemic clone cells, indicating extensive proliferation, which links telomere length and disease progression, as seen in other hematological malignancies [42,43].

According to the ALL subtype, children with B-ALL (1.90 ± 1.67 kilobases) had shorter telomeres than those with T-ALL (2.40 ± 1.08 kilobases). These findings align with those of the study by Capraro and colleagues who discovered longer telomeres in adults with T-ALL compared to those with B-ALL [14]. Consistent with our results, the telomere length values were the shortest in cases of B-ALL with the Philadelphia chromosome [40]. Another study has also provided evidence that telomere length is significantly shorter in acute leukemia patients with an aberrant karyotype than in those with a normal karyotype [15]. Conversely, a previous study indicated that low-risk B-ALL cases are characterized by long telomeres, which increases their susceptibility to poor disease outcomes [40].

According to the risk profile of children enrolled in this study, it was proven that children in the high-risk ALL group had a shorter average telomere length (1.43 ± 1.54 kilobases) compared to the intermediate-risk ALL group (2.29 ± 1.47 kilobases). Consistent with our results, a recent study has also found short telomere length values in high-risk ALL cases, suggesting that these short telomere values can precede disease progression [13]. Importantly, Jebaraj et al. [13] provided compelling evidence that leukemic clones exhibit significant telomere shortening along with chromosomal abnormalities. They discovered that individuals with 17p- and 11q-associated p53 and ATM loss, respectively, have the shortest telomeres, reinforcing the idea of the prognostic value of telomere length in ALL disease [13].

To sum up, our preliminary findings suggest that shorter telomeres are associated with an increased risk and recurrence of pediatric ALL. The preliminary results of our study focused on specific populations in the bone marrow samples collected from ALL children (lymphoblasts at diagnosis of ALL disease and at lymphocytes at remission of ALL disease). In particular, our findings indicate that lymphocytes derived from children with high-risk ALL who relapsed exhibited noticeably shorter or the same length of telomeres compared to those derived from the lymphoblasts of ALL children. As a result, our study suggests that, following the development of leukemogenesis (established leukemia), telomere shortening can accelerate genome instability, thereby increasing disease aggressiveness and resulting in poor outcomes. In this manner, our study provides preliminary data suggesting telomere length as a potential prognostic biomarker in childhood ALL, since short telomeres are markers of genomic instability.

In addition to this, previous Mendelian Randomization (MR) findings have suggested that longer telomeres of hematopoietic stem/progenitor cells might increase leukemia risk [44]. In particular, it was proposed that the genetically determined longer telomere length of hematopoietic stem/progenitor cells may increase red blood cell (RBC) and white blood cell (WBC) counts, thereby potentially increasing susceptibility to hematological malignancies, including ALL [44]. The possible explanation of the MR study stems from the fact that pre-leukemic (pre-malignant) clones with longer telomeres have a greater possibility of becoming leukemic (malignant) clones due to the accumulation of oncogenic mutations during their extensive rounds of proliferation [44].

This study has several limitations that should be mentioned. Initially, the primary limitation of this study is the small sample size. The generalizability of our findings and the statistical power to identify subtype-specific differences may be constrained by the relatively small sample size (*n* = 35). Second, a thorough assessment of telomere length dynamics during childhood ALL progression was not possible due to the preliminary nature of the qFISH data, which were only collected at diagnosis and not during remission, even though qPCR offered reliable mean telomere length values. In particular, this study provides preliminary results on the potential prognostic value of telomere length in childhood acute lymphoblastic leukemia (ALL). In addition, the comparison of telomere length between leukemic lymphoblasts at diagnosis and predominantly normal lymphocytes at remission reflects a shift in cell populations rather than longitudinal changes in the same cells, which should be considered when interpreting our results. Last but not least, telomere length as a possible prognostic marker for ALL progression and treatment response should be considered alongside other cytogenetic features of children with ALL (karyotype, disease risk). To sum up, our findings emphasize the need for further research with a larger number of patients to accurately include telomere length as a potential biomarker for childhood ALL. Ongoing studies will help confirm our initial results. Future studies with larger cohorts, longitudinal single-cell telomere analyses, and inclusion of remission samples in qFISH experiments are warranted to validate and expand upon these findings.

Several challenges also exist in terms of the methodologies used to evaluate telomere length dynamics. The advantage of quantitative polymerase chain reaction (qPCR) is that it can measure the absolute average telomere length in the cell population. The main drawback of qPCR is its inability to provide information about the mean absolute TL without considering the differences across cells and chromosomes individually [45]. Recognizing the limitations of the qPCR method, the qFISH methodology was employed, because it can detect changes across chromosomes at the single-cell level [39]. The qFISH technique is the most accurate and precise method compared to other molecular approaches for evaluating telomere length values [46,47]. The qFISH technique can also discern the median of short telomeres, as well as critical short telomeres in each chromosome at the single-cell level with precision, which are crucial for genome stability [39]. The metaphase Q-FISH approach can identify “telomere-free” ends, or very short terminal portions of chromosomes, because chromatin stability, which is linked to age-related diseases, requires the identification of telomere-free ends [46]. In addition to assessing telomere-free ends on each chromosome in each cell, the qFISH approach offers the advantage of avoiding unreliable estimations of interstitial telomere regions (ITRs) [48]. Finally, qFISH can be used to identify chromosomal fusion events and ends observed in leukemias [13,49]. Interestingly, the FISH-based method has been used to directly determine the structural rearrangements in interphase cells, with the ultimate aim of diagnosing ALL [50]. Researchers also used FISH to detect complex rearrangements and events of hyperdiploidy in the diagnosis of ALL [51].

However, several challenges need to be addressed. First, the qFISH experiments were performed on bone marrow cells derived from children with ALL at the time of diagnosis, not at the time of remission. Further research is needed to gain a more comprehensive picture of values for extremely short or extremely long telomeres, thereby offering an overall characterization of telomere length patterns. Second, one of the hallmarks of ALL is that the leukemic cells often possess a karyotype with more than 46 chromosomes, or they are either haploid or polyploid cells. However, the current analysis does not aim to clarify the molecular mechanisms responsible for the improvement in telomere shortening following treatment.

## 5. Conclusions

In conclusion, our study offers preliminary insights into the significant telomere shortening rates observed in children with ALL who have cytogenetic abnormalities or high-risk disease. Our preliminary findings suggest that telomere length differences between leukemic blasts and normal lymphocytes may serve as a potential useful prognostic marker for the evaluation of childhood ALL. However, this difference represents a cell population shift rather than longitudinal telomere recovery in the same cells. This study underscores the importance of telomere length as a potential prognostic biomarker for assessing the progression of ALL in children following treatment. Collectively, these findings highlight the necessity for further research, including a larger sample of children, to effectively incorporate telomere length as a potential reliable prognostic biomarker in the treatment of ALL. Ongoing research will confirm our initial findings.

## Figures and Tables

**Table 1:** Distribution of children by gender and age of disease, as well as average age and current age of children.

Demographic	Value	Count (*n*)		%
Gender	Male	22		62.9
	Female	13		37.1
Age	<1	0		0
	1–9.9	28		80
	>10	7		20
	Mean ± SD		Minimum–Μaximum age	
Age of onset	6.4 ± 4.1		1.7–18.0	
Current age	11.8 ± 3.5		5.8–19.3	

SD: standard deviation.

**Table 2:** Classification of children with acute lymphoblastic leukemia (ALL) based on risk of relapse stratification according to ALLIC-BFM 2009 treatment protocol criteria and karyotype at diagnosis and relapse.

Clinical Characteristics	Value	*n*	%
Risk stratification	High Risk	12	34.3
	Intermediate Risk	23	65.7
Relapse	Νο	32	91.4
	Yes	3	8.6
Karyotype	46 Chromosome	21	63.6
	46+ Chromosome	12	36.4

Chr: chromosome.

**Table 3:** The mean telomere length (TL) of patients in kilobases (Kbases) according to the type of acute lymphocytic leukemia (ALL), their risk stratification and karyotype.

		TL (Kbases)			
		Mean	SD	Median	*p*
Category of ALL	B-ALL	1.900	1.670	1.780	0.570
	T-ALL	2.400	1.080	2.500	
Risk stratification	High risk	1.430	1.540	1.630	0.118
	Intermediate risk	2.290	1.470	2.150	
Karyotype	46 Chromosome	1.610	1.400	1.670	0.030
	46+ Chromosome	3.020	1.780	2.430	

## Data Availability

The data presented in this study are available on request from the corresponding author upon reasonable request.
